# Astaxanthin as a King of Ketocarotenoids: Structure, Synthesis, Accumulation, Bioavailability and Antioxidant Properties

**DOI:** 10.3390/md21030176

**Published:** 2023-03-13

**Authors:** Anagha Nair, Ankesh Ahirwar, Shashikala Singh, Reeta Lodhi, Aishwarya Lodhi, Anshuman Rai, Dipak A Jadhav, Harish, Sunita Varjani, Gurpreet Singh, Justine Marchand, Benoît Schoefs, Vandana Vinayak

**Affiliations:** 1 Diatom Nanoengineering and Metabolism Laboratory (DNM), School of Applied Science, Dr. Harisingh Gour Central University, Sagar 470003, India; 2 Department of Biotechnology, School of Engineering, Maharishi Markandeshwar University, Ambala 133203, India; 3 State Forensic Science Laboratory, Madhuban 132037, India; 4 Department of Environmental Engineering, College of Ocean Science and Engineering, Korea Maritim and Ocean University, Busan 49112, Republic of Korea; 5 Department of Botany, Plant Biotechnology Laboratory, Mohanlal Sukhadia University, Udaipur 313001, India; 6 School of Energy and Environment, City University of Hong Kong, Hong Kong 999077, China; 7 Sustainability Cluster, School of Engineering, University of Petroleum and Energy Studies, Dehradun 248007, India; 8 Metabolism, Bioengineering of Microalgal Metabolism and Applications (MIMMA), Biology of Organisms, Stress, Health and Environment, Le Mans University, IUML—FR 3473 CNRS, 72000 Le Mans, France

**Keywords:** astaxanthin, antioxidant, accumulation, carotenoids, synthesis, stress

## Abstract

Astaxanthin (3,3-dihydroxy-β, β-carotene-4,4-dione) is a ketocarotenoid synthesized by *Haematococcus pluvialis/lacustris*, *Chromochloris zofingiensis*, *Chlorococcum*, *Bracteacoccus aggregatus*, *Coelastrella rubescence*, *Phaffia rhodozyma*, some bacteria (*Paracoccus carotinifaciens*), yeasts, and lobsters, among others However, it is majorly synthesized by *Haematococcus lacustris* alone (about 4%). The richness of natural astaxanthin over synthetic astaxanthin has drawn the attention of industrialists to cultivate and extract it via two stage cultivation process. However, the cultivation in photobioreactors is expensive, and converting it in soluble form so that it can be easily assimilated by our digestive system requires downstream processing techniques which are not cost-effective. This has made the cost of astaxanthin expensive, prompting pharmaceutical and nutraceutical companies to switch over to synthetic astaxanthin. This review discusses the chemical character of astaxanthin, more inexpensive cultivating techniques, and its bioavailability. Additionally, the antioxidant character of this microalgal product against many diseases is discussed, which can make this natural compound an excellent drug to minimize inflammation and its consequences.

## 1. Introduction

**Figure 1:**
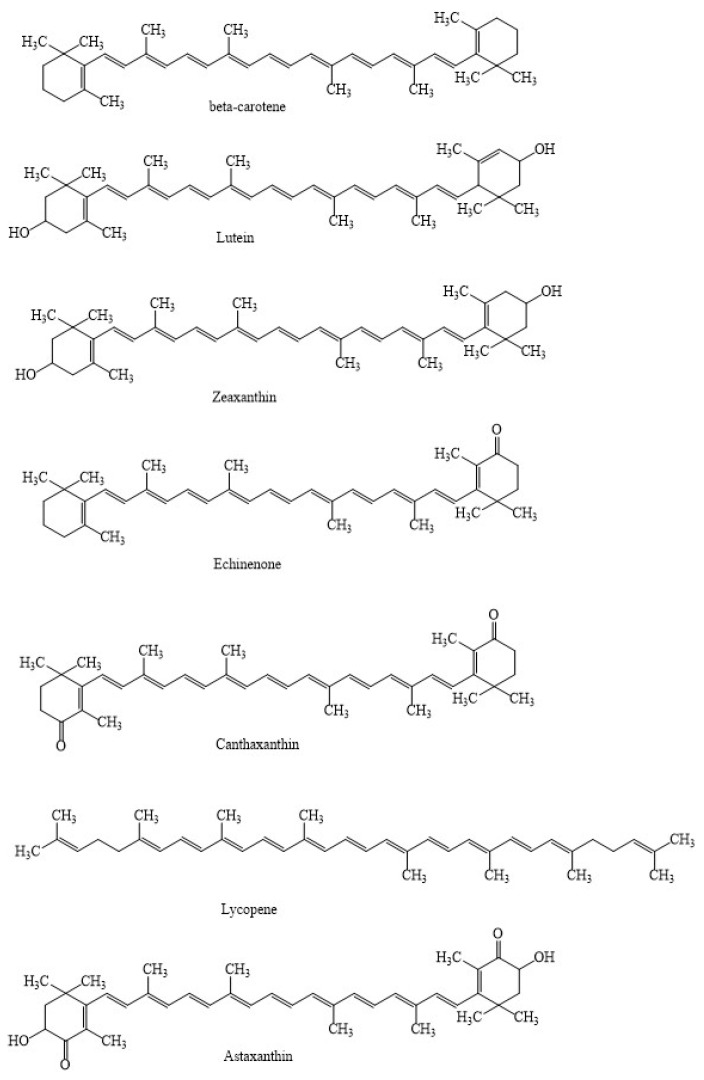
Structure of different carotenoids.

Among existing carotenoids, about 600 different types are biosynthesized in plants, mosses, algae, bacteria, and fungi [1,2,3]. Most carotenoids are composed by 40 carbon atoms joined by double bonds (Figure 1) [4,5,6]. These have further been grouped into carotenes and xanthophylls [7,8,9]. Carotenes are composed of only carbon and hydrogen atoms, whereas xanthophylls are oxygenated derivatives which may have hydroxy groups (e.g., zeaxanthin), keto groups (e.g., canthaxanthin) or combinations of groups (e.g., astaxanthin, fucoxanthin, violaxanthin) [10,11,12]. 

Among carotenoids, the ketocarotenoid astaxanthin (3,3′-dihydroxy-β, β′-carotene-4,4′-dione) occupies a particular place because of its intense red color and antioxidant properties that are ca. 20–50 times higher than synthetic astaxanthin [13], but the antioxidant properties of free and esterified astaxanthin are still debated [14]. These unique properties of natural astaxanthin are among the primary reasons of its high value in the market which ranges from $6000 to $7150 per kg and the commercial market for astaxanthin has shown a great potential and is estimated to grow up to 3.4 billion by 2027. Nevertheless, cheap chemical astaxanthin meets 95% of the market demand [15,16,17]. Considering this price difference, replacing synthetic astaxanthin by natural astaxanthin is not cost effective and therefore requires strategies and techniques to increase astaxanthin production for cutting down the cost of its production. This includes photobioreactors and economically efficient downstream processing [18] and/or reconsidering astaxanthin sources besides *Haematococcus lacustris*, which is the major producer of astaxanthin (about 4% of dry weight) and is already cultivated at an industrial scale [19,20,21,22].

Astaxanthin was discovered in lobsters (*Astacus gammarus*) by the Nobel laureate Richard Kuhn [23]. Since this pioneer work, astaxanthin has been found to be naturally synthesized by land plants (*Adonis annua:* [24] and *Adonis aestivalis*: Egger and Kleinig [25]), microalgae (*Haematococcus laccustris*, *Chromochloris zofingiensis* (formerly *Chlorella zofingiensis*: Fucikova and Lewis [26], *Chlorococcum* sp.: Kopecky, et al. [27], *Bracteacoccus aggregatus* [28], *Coelastrella rubescence* [29], *Chlamydomonas nivalis*: Viala [30], yeasts (*Phaffia rhodozyma*) and some bacteria eg. *Paracoccus carotinifaciens*, a motile aerobic Gram-negative bacterium [31,32].

**Figure 2:**
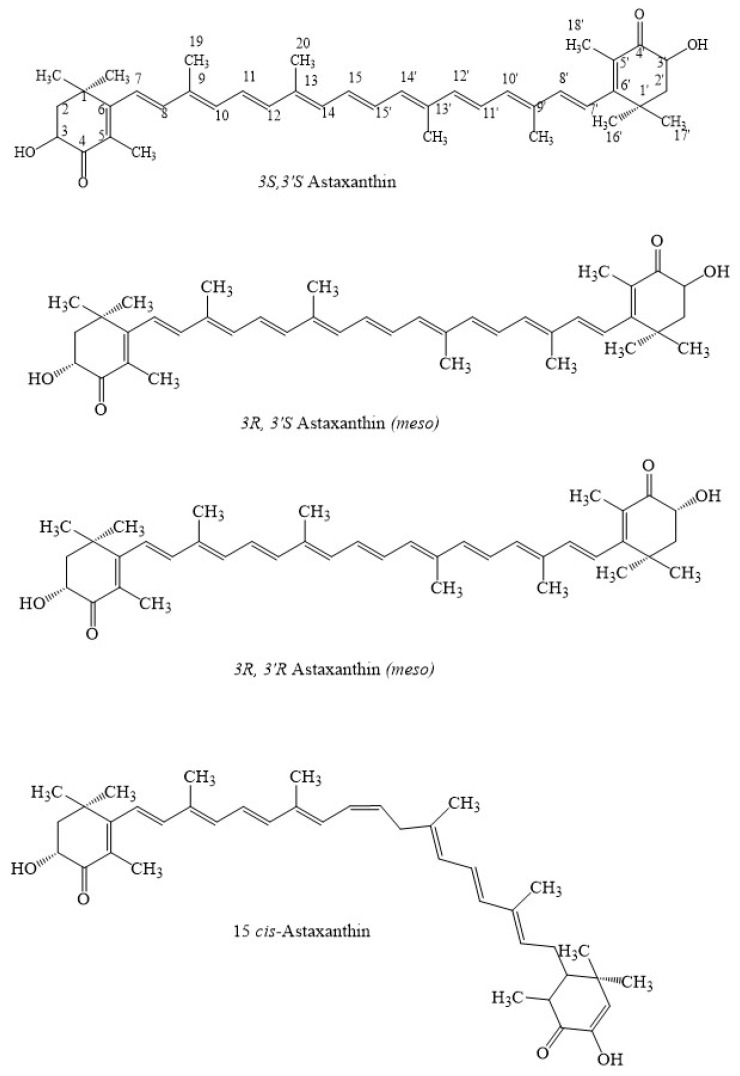
Different isomers of astaxanthin.

Astaxanthin, as with other carotenoids, is characterized by a polyene system allowing the molecules to exist in *cis*- and *trans*-isomeric forms. The *cis*-configuration is comparatively less stable than the *trans*-isomeric form [6]. Most of the carotenoids found in nature exhibit a *trans*-configuration. In addition, astaxanthin has chiral carbon at the C3 and C3′ positions, allowing the existence of two enantiomers (3*R*, 3′*R* and 3*S*, 3′*S*) and one *meso*-configuration (3*R*, 3′*S*) (Figure 2). Another type of chemical modification of astaxanthin is esterification with one or two fatty acids [33]. Altogether, these chemical particularities confer to the carotenoids typical molecular configurations that may impact their light absorbing properties [34], chemical stability and bioavailability [35]. For instance, mono-esterified astaxanthin with unsaturated fatty acids and a short chain is rapidly hydrolysed in the human digestive system [36], indicating that free astaxanthin bioavailability is higher in comparison to mono-esterified and diesterified astaxanthin.

**Figure 3:**
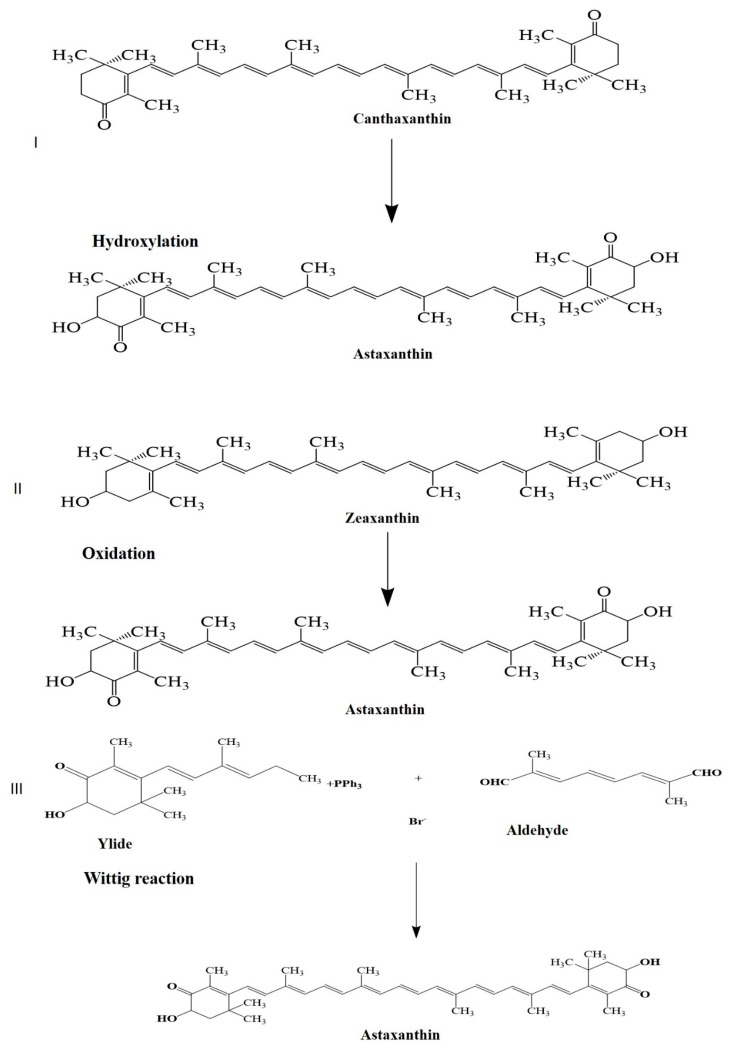
Possibilities of chemical synthesis of astaxanthin by (**I**) Hydroxylation; (**II**) Oxidation; (**III**) Wittig reaction.

Astaxanthin from *Haematococcus lacustris* is mainly the 3*S*, 3′*S* isomer which is 70% monoesterified, 25% diesterified and 5% free form with 3′-OH group modification [37], whereas that from *Phaffia rhodozyma* is 3*R*, 3′*R* unesterified astaxanthin due to its biosynthesis [38]. Astaxanthin from *Paracocuss carotinifacience* is 100% free form with 3,3′-OH group modification [39]. Note that synthetic astaxanthin is produced from petrochemical products [40] via a multistep process that includes hydroxylation of canthaxanthin, oxidation of zeaxanthin and the Wittig reaction [36] (Figure 3). Synthetic astaxanthin is a mixture of stereoisomers, 1(3*R*, 3′*R*), 2(3*R*, 3′*S*), and 1(3*S*, 3′*S*) nonesterified forms [41,42]. 

Only natural astaxanthin is considered safe for human use and consumption. Due to its multiple applications in pharmaceutical, nutraceutical, cosmetics, dietary and aquaculture, astaxanthin is produced naturally by many companies due to its rapidly growing list of attributes [43]. Chemically synthesized astaxanthin is not approved for direct consumption because of its chemical residues, which are not appropriate for humans [44] although they are recommended for aquaculture [45].

This review discusses the chemical structure and synthesis of carotenoids, mainly astaxanthin. It also describes cultivation strategies, primarily of *Haematococcus lacustris* for astaxanthin production. The review throws light on accumulation, harvesting, and bioavailability of astaxanthin. The pathophysiology of diseases caused by poor immunity, and the role of astaxanthin in regulating the immune responses is described. Additionally, a special focus is on the ability of astaxanthin to reduce inflammation and reactive oxygen species (ROS), resulting in several other health benefits. 

## 2. Cultivation and Accumulation of Astaxanthin in *Haematococcus lacustris*

**Figure 4:**
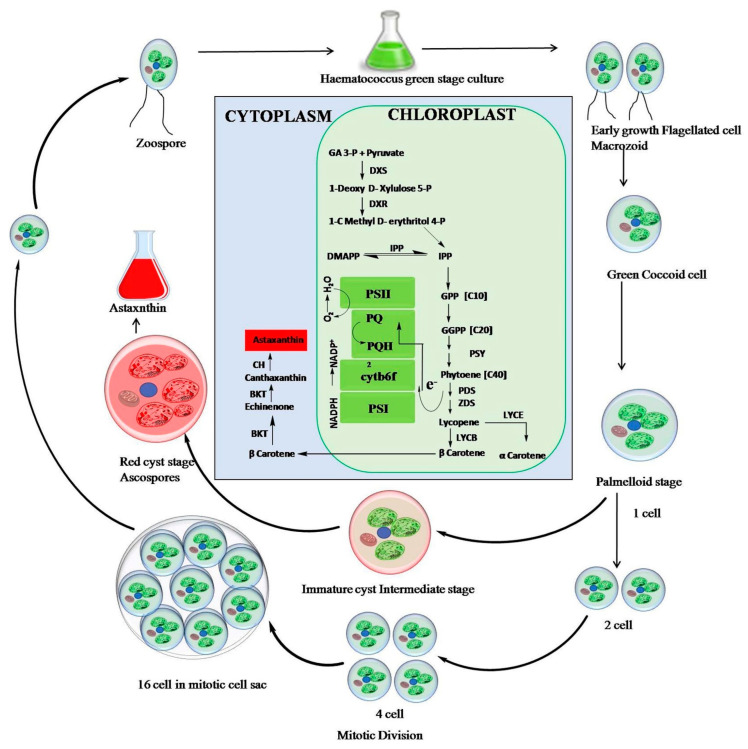
Biosynthetic pathway of astaxanthin synthesis from *Haematococcus*. Reproduced with permissions from [17].

*Haematococcus lacustris* has three different life stages, i.e., macrozooids, microzooids, coccoid vegetative cells and palmella or haematocyst. Green macrozooids are found in a nonstressful environments, i.e., when all the required nutrients are available for multiplication, and is mostly asexual [46]. When one environmental condition becomes limiting, macrozooids are transformed to palmella and start to accumulate astaxanthin in the cytoplasm, requiring deep metabolic reorientation. At the same time, cells stop diving, enlarge and enter the red cyst stage, enriched in astaxanthin [47]. Figure 4 shows the morphological, biochemical and gene pathway stages for astaxanthin synthesis, and the mechanism [17,48]. From the carotenoid point of view, green macrozooids contain the typical carotenoids of green microalgae, i.e., β-carotene, lutein, violaxanthin and neoxanthin, whereas at cyst stage they have 90% astaxanthin, along with its mono and di esters [49].

### 2.1. Biochemical Pathway

In green microalgae the keto group in carotenoids is at the fourth position of the ionone rings. In contrast to β-carotene that accumulates in the plastoglobuli in the *Dunaliella bardawil* chloroplast matrix [50], astaxanthin accumulates in cytoplasmic lipid globules of *Haematococcus lacustris* [6]. 

In the biosynthetic pathway for astaxanthin synthesis (Figure 4), Isopentenyl pyrophosphate (IPP) is the precursor molecule [51] and can be produced via two pathways, i.e., the mevalonate (MVA) and methyl erythritol 4-phosphate (MEP) pathways. However, in *Haematococcus* it is produced via the MEP pathway only [52]. IPP is coded by two enzymes *ipi1* and *ipi2*. IPP undergoes a set of reactions involving isomerization, condensation and desaturation yielding β-carotene. The β-carotene is then transferred from chloroplast to cytoplasm for the formation of astaxanthin in the presence of enzyme Cyt P450- β-carotene hydroxylase (CRTR-b), whose ketolase activity is present only in the cytoplasm, resulting in upregulation of *crtr-b* gene [53]. This is accompanied by an active tricarboxylic acid (TCA) cycle [54,55]. 

### 2.2. Wastewater as Growth Medium for Haematococcus

In the recent past, several companies have taken steps in the direction of production of natural astaxanthin, but the technology is still in its infancy with regard to bringing down the cost of production [56]. Although various media recipes are recommended, such as Bold Basal Medium (BBM), Blue Green medium (BG−11), the National Institute for Environmental Studies medium (NIES), Tris acetate phosphate (TAP) and the Optimal *Haematococcus* Medium (OHM) [57,58] for the growth of *Haematococcus*, none are economical for large-scale cultivation. On the offset, the cultivation can be made economical if *Haematococcus lacustris* is grown in pretreated wastewater since it has ability to remediate wastewater by utilizing the basic nutrients such as nitrates, phosphates and trace metals that are present in abundance in the wastewater [59]. The microalgae reduce the nitrates, phosphates, ammonia, sulphates and reduce the chemical oxygen demand (COD), while simultaneously producing biomass and various value added products [60,61]. Kang C.D et al., 2006 [62] found that *Haematococcus* grown in primary treated sewage and pretreated piggery wastewater diluted four times (filtered via 0.2 µm pores and pH set to 7.5) completely removed nitrates at 42.4 mgL^−1^ and 469 mgL^−1^,respectively, phosphates at 2.6 mgL^−1^ and 98.7 mgL^−1^, respectively, as well as other salts and COD. The astaxanthin accumulated in primary treated sewage and pretreated piggery wastewater was 39.7 mgL^−1^ and 83.9 mgL^−1^, respectively. In another study, pretreated potato wastewater was used as the growing nutrient medium for *Haematococcus lacustris*. It was found that astaxanthin accumulation in *Haematococcus* cells grown in potato wastewater reached to 24.5–27.9 mgg^−1^ in 3 days. This was much higher than for control cells, which showed only 14.7 mgg^−1^ astaxanthin accumulation in 12 days [63]. This is quite encouraging, as this simultaneously produced methane while remediating high strength organic wastewater with efficient removal of COD, phosphates, nitrates, and ammonia.

### 2.3. Growth of Haematococcus in Photosynthetic Microbial Fuel Cells

**Figure 5:**
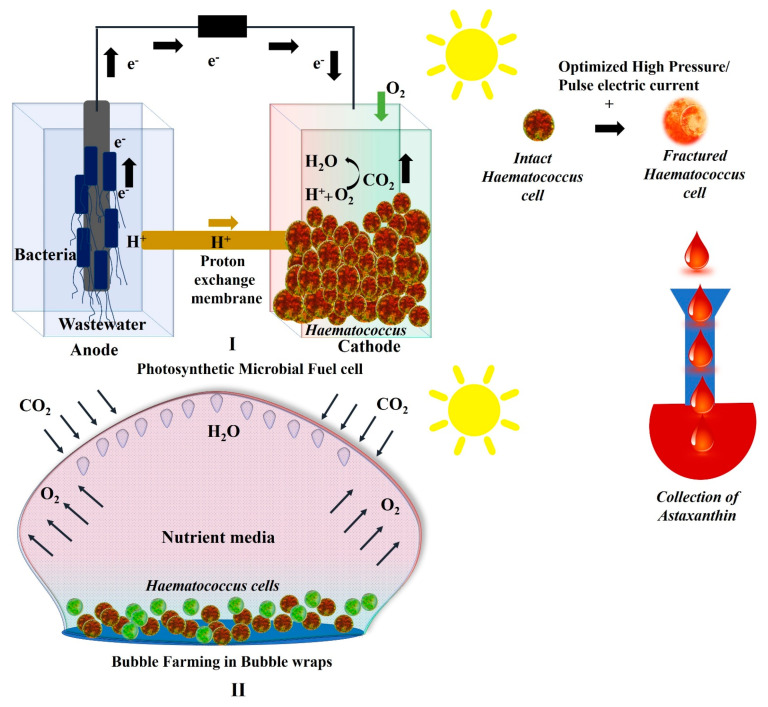
Economical cultivation of *Haematococcus lacustris* for astaxanthin: (**I**) Photosynthetic microalgal microbial fuel cell with *Haematococcus lacustris* and cells showing their harvest to astaxanthin on being applied optimized high pressure/pulse electric current. (**II**) Bubble farming in a plastic bubble wrap showing *Haematococcus lacustris* cells being grown in it with no water loss and sufficient exchange of gases.

The latest technique for remediating wastewater is the use of microbial fuel cells [64]. Microbial fuel cells are biochemical devices in which electrons are produced by anaerobic bacteria during the process of degrading organic matter in wastewater at the anode. The redox reactions occurring at the anode and cathode are separated by a proton exchange membrane and connected to a resistor [65]. The process produces electricity while remediating wastewater pollutants at the anode. Further, if the electron acceptors at cathode are replaced by living microalgae, this is known as a photosynthetic microalgal microbial fuel cell [66,67]. Thus, if *Haematococcus lacustris* is used as a cathode substrate in a microbial fuel cell it produces free oxygen. Hence, in this process, the wastewater is remediated and bioelectricity is produced, besides the production of value-added products in the form of biomass and pigments [68]. Such kind of microbial fuel cell that hybridize with an algal photobioreactor separated by a proton exchange membrane such as a clay plate which are economical it can reduce the cost of astaxanthin production when upscaled [69,70,71]. Although in a microbial fuel cell, only electrogenic microbes are used at the anode, recently Khan et al. 2022 [72] found that diatoms (brown microalgae) are electrogenic when used at the anode due to NOX^-^ ions produced in diatoms for iron reduction. The MFC thus produces electricity (12.6 mWm^−2^), lipids (22.31 μg mL^−1^ on 15th day) and pigments (4.890 μg mL^−1^ on 15th day). Further, the dead diatom frustules/biomass act as polyabsorbents for absorbing pollutants from the wastewater [73]. Similarly a schematic representation of photosynthetic MFC with *Haematococcus* at the cathode, and harvesting of its cells for astaxanthin can be seen in Figure 5.

### 2.4. Growth of Haematococcus in Plastic Bubble Wraps

The harvesting of *Haematococcus lacustris* for large scale production of astaxanthin is generally carried out in open raceways, i.e., closed photobioreactors at large scale, but it has its drawbacks. The open raceway often gets contaminated has irregular irradiation of light, and requires a regular supply of media and water, whereas closed photobioreactors requires expensive photobioreactors, a electricity supply, and maintenance of pH, CO_2_ and temperature, which is not economically viable [74]. However, the concept of bubble farming has been introduced in which algae are grown in plastic bubble wraps discarded from the plastics industry [75] (Figure 5II). In recent work on diatoms cultivated in different types of plastic bubble wraps, it has been established that low density polypropylene bubble wraps proved to be the best closed-chamber, air-filled photobioreactor, allowing the exchange of air but inhibiting water evaporation, and avoiding cell contamination. The cells grew exponentially and survived for months without losing their water content compared to cells kept in an open container. The chlorophyll content in bubble wraps reached to 3.34 μg mL^−1^ compared to 0.735 μg mL^−1^ in the control on 40th day and after [75]. The growth of *Haematococcus* was also tested in small scale plastic bubble wraps (Volume 160 µL in a bubble wrap of diameter 0.25”) and showed a similar pattern of gaseous exchange, no contamination, and multiplication of cells with no loss of water [75]. In addition to bubble wraps, all closed plastic containers made up of low density polypropylene, or any other plastic material that allows exchange of gases, inhibits water loss, and is transparent to light, are suitable for cultivating microalgal cells for their value-added products [76]. 

### 2.5. Stress Factors to Induce Astaxanthin Production in Haematococcus

The effect of carotenogenesis depends upon environmental factors such as temperature and light [77]. Optimum temperature promotes growth of cells, while light enhances cell multiplication during the juvenile stage of cells, and high light favours astaxanthin production in *Haematococcus lacustris* [49]. 

*Haematococcus* can be cultivated in two stages, first cultivating for the green stage, and then shifting the cells towards stress environment to simulate the red stage for astaxanthin production. There are many factors which may result in increasing astaxanthin content in *Haematococcus lacustris* in vitro, which include both physical and chemical factors. There have been intense studies on various chemicals to increase astaxanthin yield, and the genes responsible for its upregulation have been monitored via transcriptomics. High light and nitrogen deficiency are the most common factors to induce the shift from green cell stage of *Haematococcus lacustris* towards the red cell stage for astaxanthin synthesis [78]. Further it is the stress environment that increases reactive oxygen species level in the cell environment of *Haematococcus* that is responsible for increasing the astaxanthin level [79]. In addition, gamma amino butyric acid (GABA) maintains the stress tolerance of the cells under a stress environment [80]. *Haematococcus* cells showed the highest biomass (1.65 g L^−1^), of astaxanthin (3.86 mg L^−1^ d^−1^) and lipids (ca. 55.11%) when treated with 0.25 mM GABA. Additionally phytohormones and chemicals enhance astaxanthin synthesis and accumulation [54]. In addition, polyamines, such as putrescine, spermidine and spermine play an important role in regulating stress. For instance, melatonin and putrescine are stress signal chemicals that are inexpensive and improve astaxanthin synthesis in *Haematococcus*. These chemicals can be an appropriate choice for reducing culture and cultivation costs for astaxanthin production. Thorough research of all the chemicals and factors that enhance astaxanthin production in *Haematococcus*, along with their transcriptomic data, is necessary to analyze the upregulated genes and downregulated genes as shown in Table 1. However, there are still many unexplored chemicals and factors for which transcriptomics gene studies are pending. In brief, to make the whole process of astaxanthin production and accumulation cost-effective, different factors influencing the accumulation of astaxanthin and genes responsible, with their complete transcriptomic data, need to be explored.

## 3. Astaxanthin Harvesting and Its Bioavailability

### 3.1. Harvesting of Haematococcus for Astaxanthin

**Figure 6:**
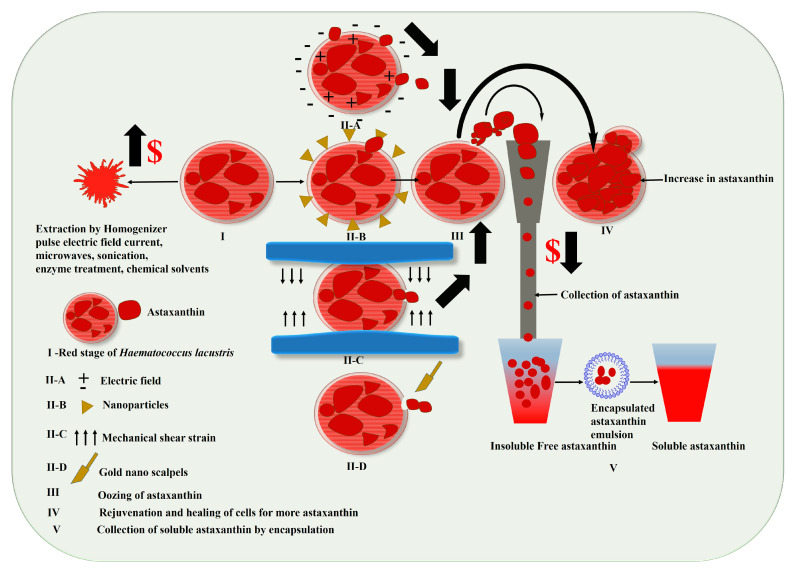
Harvesting techniques showing better astaxanthin synthesis, accumulation without cell lysis, and its bioavailability, where **I**: Intact *Haematococcus* rich in astaxanthin.; Cell wall fracturing by **IIA**: Electrolysis; **IIB**: Nanoparticles; **IIC**: Mechanical shear strain; **IID**: Gold nano scalpels; **III**: Haematocccus cell oozing astaxanthin without cell lysis; **IV**: Rejuvenation and healing of *Haematococcus* cells for more asataxanthin and **V**: Collection of soluble astaxanthin by encapsulation.

The rigid cell wall of *Haematococcus lacustris* (~2.2 µm) is made up of algaenan, biopolymers and thick polysaccharides [96,97]. Since, cell disruption in downstream processes such as pulse electric field current, microwaves, sonication, enzyme treatment, chemical solvents, high pressure homogenizers and bead millers, are expensive at large scale, they tend to increase the cost of extraction of astaxanthin [98,99] (Figure 6I). However, if among these, an economically viable technique is optimized that can lead to harvesting of astaxanthin from the cells, astaxanthin can become an important ingredient in all affordable diets and supplements [100,101]. A recent study has shown that on applying a mechanical strain, *Haematococcus* cells when experienced shear stress, oozed astaxanthin in a microfluidic chamber [102]. A microfluidic chamber with 65 micropillars with six different heights was used for selecting the *Haematococcus* cells put under mechanical stress. The gap between the micropillars was 10 µm to allow the young, flagellated cells to flow through. The height of drain channel was kept at 5 µm to prevent it from draining off. Mechanical stress was applied by loading the cells in culture chambers of different sizes ranging from 5 µm to 70 µm. Analysis done by Raman spectroscopy, showed that the amount of astaxanthin accumulated in different culture chambers inducing mechanical stress was maximum in a chamber with a height of 15 µm. This is similar to earlier reports on diatoms where mechanical shear strain generated by resonance energy in a microfluidic channel caused diatoms to ooze oil without lysing [103]. Studies have also shown that nanotechnology employing nanoparticles and laser light irradiation can electroporate the cell wall of *Haematococcus*. It has been observed that α-quartz nanoplates (NPLs) in combination with an ethyl-3-methylimidazolium (Emim) ionic liquid helps in extracting astaxanthin from *Haematococcus* at room temperature [104]. It was observed that extraction by ionic liquid alone was much less effective than the combination of ionic liquid and α-quartz NPLs. It was observed that when 90-days-old *Haematococcus* cells were treated with ionic liquids such as thiocyanate, diethylphosphate, HSO_4_, and Cl^−^ ions, the extraction of astaxanthin was quite low at about 9.6–14.2%. However, when these ionic liquids were combined with α-quartz NPLs to treat 90-days-old *Haematococcus* cell, it was found that the extraction efficiency increased and was highest for the ionic liquid HSO_4_ (80.7%). In a study by Praveen Kumar et al., 2015 [105], a gold nanoscalpel (Au-NS) (300 nm thick and 1–3 µm of width) synthesized by a chemical vapor transport method, and mounted on a three dimensional piezoelectric stage incised single cell of *Haematococcus* kept in an agarose microwell, showed 2 times more accumulation of astaxanthin. This milking, or harvesting, by Au-NS on *Haematococcus* cells leads to regular harvesting of astaxanthin without cell lysis. Thus, different techniques such as electroporation, use of nanoparticles, mechanical shear strain and nanoscalpels, may be considered for harvesting astaxanthin from *Haematococcus*, as shown in Figure 6IIA–D and III. The fractured cells are followed by healing and reaccumulation, compared to a normal cell (Figure 6IV).

### 3.2. Astaxanthin Bioavailability

Astaxanthin is a super antioxidant molecule having significant biological functions and the ability to cross the blood, brain and retinal barriers [106]. This depends upon its absorption, transport, and its metabolism mechanism in humans. Among the 600 types of identified carotenoids, around 50 carotenoids occur naturally in the food, and only about 20 carotenoids have the capability to be absorbed in the animal intestine and further distributed to tissues [107]. The absorption of astaxanthin in the human digestive system is further dependent upon bile salts, pH, and glycine/taurine ratios. The uptake and absorbance of carotene is four-fold higher in the presence of bile salts and as a part of a fatty food meal. Briefly, astaxanthin is absorbed with fatty acids into the intestinal epithelium via a passive diffusion mechanism. Further, astaxanthin combines with secretions from the spleen, resulting in the formation of micelles in the intestine, which is partially engrossed by intestinal mucosal cells. These cells deliver the astaxanthin to the liver, and in the liver, it mixes with lipoproteins and gets transported to the body tissues by circulation. Additionally, one of feature of astaxanthin molecules is their insertion into the lipid bilayer without degrading the cell membrane structure, thereby shielding the redox state and functional integrity of mitochondria [108]. The bioavailability of astaxanthin can be estimated by its chemical nature, such as its degree of esterification, geometrical isomer form, and optical stereoisomer form. The oral availability of astaxanthin, however, depends on its time of consumption before and after meals in non-smokers and smokers. It was found that the bioavailability of astaxanthin was higher at 7.219 ± 3.118 µg h/l in the after-meal group, and 2.968 ± 959 µg h/l in the before meal-group in non-smokers, and higher than in smokers before (*p* < 0.05) and after meals (6.468 µg h/l) [109]. Nanoencapsulation of astaxanthin improves its bioavailability as well as its bioabsorption by dispersing its insoluble bioactives [110,111]. Figure 6V shows the better solubility of encapsulated nano emulsion of astaxanthin over free astaxanthin. It has found by Edelman R et al. [112] that delivery by potato protein (PP) astaxanthin nanoparticles enhanced the solubility and bioavailability of astaxanthin compared to astaxanthin alone. The encapsulated astaxanthin did not degrade at all in an in vitro simulated digestion model in humans, whereas unencapsulated astaxanthin degraded in 120 min, and at the end of digestion (240 min). The fact that PP astaxanthin did not degrade was due to extremely low pH (2.5) and the property of protease inhibitors in the PP, which inhibited trypsin and chymotrypsin degradation. Although there are many encapsulation techniques, since astaxanthin is thermolabile, encapsulation methods that avoid heating are preferred. Since astaxanthin has properties to block cell cycle progression leading to apoptosis, a new method of incorporating astaxanthin with poly(lactic-co-glycolic acid) (PLGA)-encapsulated astaxanthin and processed with ultrasonically treated broccoli-derived extracellular vesicles (BEV) to form astaxanthin@PLGA@BEV has shown better anti-cancer property against human colon cancer cells (HT-29) than in vivo astaxanthin [113]. Additionally, surface polymers such as arabic gum improve encapsulation efficiency, and spray drying, ionotropic gelation, nanoprecipitation, emulsification-evaporation have shown to be cost-effective ways of encapsulating astaxanthin [114,115,116,117,118]. 

## 4. Anti-Oxidative Properties of Astaxanthin versus Other Carotenoids

Carotenoids have a potential role against viral and bacterial diseases by blocking the cellular angiotensin converting enzyme (ACE2) receptor, regulating inflammation, and modulating the peroxisome proliferator activator response (PPARγ) expression [119]. They thus have anti-inflammatory properties that help in decreasing the oxidative stress checking the invasion of cytokine storm. An adequate dose (4–12 mg/day) of astaxanthin for 3 months serves against many common infections in healthy people (Lu et al., 2022). Astaxanthin is reported to have higher antioxidant activity compared to several other carotenoids such as lycopene, lutein, zeaxanthin, α-carotene and β-carotene in human studies [120]. Astaxanthin from *Haematococcus lacustris* fed to rats had increased antioxidant effects [121]. Astaxanthin is completely digested by lipoprotein lipase found on the surface of cells and excreted by liver. The safe dosage for human consumption of astaxanthin has been increased from 4 mg/day to 12 mg/day by the European Food Safety Authority and the US Food and Drug Administration respectively, provided the intake is within 30 days [122]. A study in ochratoxin (OTA)-induced lung injury in a mouse model showed that astaxanthin protected the organs from oxidative damage and inflammation via the nuclear factor (Nrf2/NF-κB) pathway, which is related to maintaining innate immunity [123]. Carotenoids also have an overall effect in reducing the serum concentration of C-reactive protein (CRP) and interleukin -6 (IL-6) produced in response to infections. Amongst available carotenoid supplements, astaxanthin exhibits the most promising anti-inflammatory effects in humans [119].

**Figure 7:**
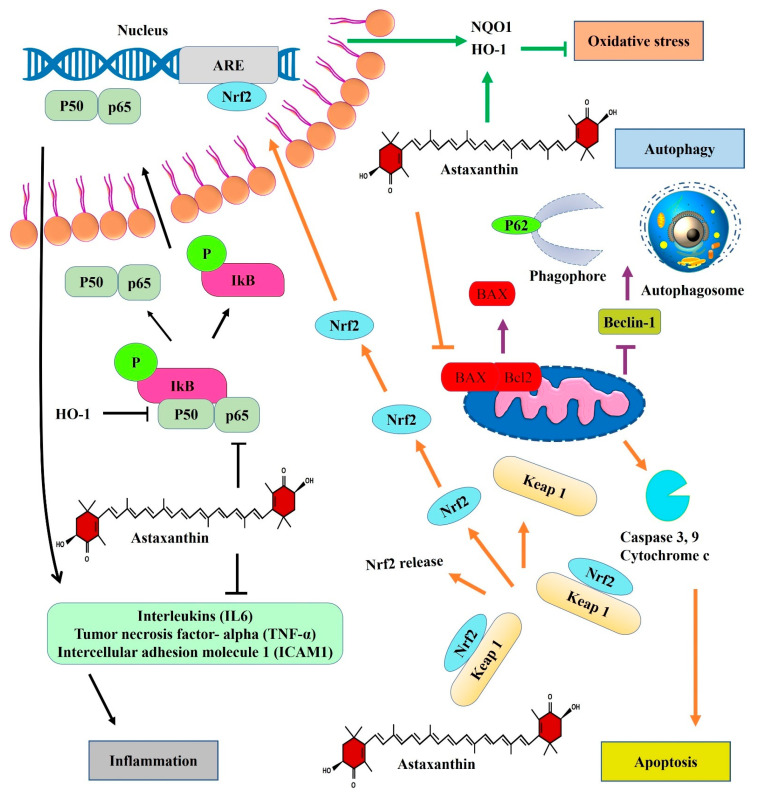
Different mechanism of astaxanthin action inside human body. Note: *Nrf2: Nuclear factor erythroid 2-related factor 2; Keap: Kelch-like ECH-associated protein 1; HO-1: Heme oxygenase-1 (HO-1); BAX: Bcl-2 Associated X-protein; IL6: Interleukin 6; TNF-ALPHA: Tumour Necrosis Factor alpha; ICA: intercellular adhesion molecule 1; IKB: inhibitor of nuclear factor kappa B; ARE: Antioxidant response element; NQ01: NAD(P)H quinone dehydrogenase 1*.

Astaxanthin is not a known antiviral compound, but it has shown properties to suppress viral infections due to its antioxidant and anti-inflammatory effects. It plays a key role in regulating the cytokine storm responsible for inflammation. Astaxanthin based drugs inhibit viruses and other microbes by stopping their replication, gene expression and multiplication in host cells. Research reports have shown that astaxanthin has anti-inflammation, antioxidative, autophagy and anti-apoptosis effects in stopping pathogenesis (Figure 7). This starts with a change in nuclear factor (NF-κB) and interferon regulatory 3 (IRF3) of the host cell nuclei. It further leads to changes in the activity of transcription factors that in macrophage polarization cause expression of C-reactive proteins (CRP), ferritin, D-dimers, IFN-1, and pro-inflammatory cytokines such as tumor necrosis factor (TNF-α), IL-6, and monocyte chemoattractant factor (MCP-1). The increase in influx of these cytokines causes increase in mast cells, neutrophils and macrophages, which cause disruption in first line of defense against the virus leading to inflammation cytokine storm syndrome (CSS) and macrophage activation syndrome (MAS), as well as the disruption of lung function [124,125]. Astaxanthin has the capability to reduce oxidative stress and free radicals 65 times more effectively than vitamin C, 54 times more than carotene, and about 100 times more than tocopherol [126]. It has further shown inhibitory action on macrophage activation, phosphorylation of nuclear factor kappa B (NF-KB), Janus kinase, signal transducer and activator transcription (STAT), interleukin (IL)-6, IL-1β, cyclooxygenase- 2 and TNF-α factors [31,127,128,129].

The radical scavenging effect of α-tocopherol a fat-soluble antioxidant, is taken as a standard in comparison with β-carotene, which has lesser antioxidant properties. However, on comparing the antioxidant properties of canthaxanthin and astaxanthin in comparison with α-tocopherol and β-carotene investigated in a rat live microsome membrane exposed to 25 mMAAPH, 2,2′-azobis (2-amidinopropane); chelated Fe^3+^ and NADPH, induced free radical chain oxidation and formation of hydroperoxide formation (malondialdehyde (MDA)), it was observed that 2.3 nmol/mg protein of astaxanthin inhibited MDA and its hydroxide formation, whereas astaxanthin and canthaxanthin together inhibited at a rate greater than α-tocopherol alone. On the other hand, carotene at 10 nmol/mg protein was ineffective in inhibiting the MDA formation [130].

Astaxanthin is known to display greater biological activity compared to other anti-oxidants due to its ability to bind within the lipid bilayer of the cell membrane [131]. The polyene chain structure of astaxanthin traps the radicals inside the membrane of a cell, while the terminal ring of astaxanthin eliminates radicals from within the cell membrane as well as from its surface (Jinu & Mohan Chandra, 2021), as seen in Figure 7.

Furthermore, astaxanthin is reported to be upregulated in the irradiated cells of glutathione peroxidase 1, catalase, antioxidative enzymes, Nrf2 targeted proteins and heme oxygenase-1 (HO-1) (Jinu & Mohan Chandra, 2021). It blocks formation of ROS and triggers the expression of oxidative stress-responsive enzymes, including HO-1, which is further regulated by transcription factors such as Nrf2, reported to be activated by astaxanthin, thus protecting against oxidative stress in mice (Kubo et al., 2019).

Increased levels of ROS, as partially reduced oxygen metabolites, is strongly correlated with inflammation, oxidative injury, and viral infection and replication. It is speculated that regulating ROS level in virus-infected patients could be effective against hyperinflammation, reduce exacerbation of the immune system and protect tissues from oxidative injury, along with suppression of viral replication (Qin et al., 2020). The chemical properties and the molecular structure of astaxanthin explains its greater antioxidant activity. The natural form of astaxanthin with the all-*trans* isomer has better antioxidative properties than other carotenoids such as β-carotene, zeaxanthin, canthaxanthin, vitamin C, and vitamin E. Previous in vitro and in vivo studies on all-*trans* natural astaxanthin isomers have demonstrated its antioxidant effect and strong inhibition of lipid peroxidation (Liu & Osawa, 2007). In a study conducted by Liu et.al [132] in human neuroblastoma SH-SY5Y cells, it was reported that the *cis* astaxanthin (9-*cis* isomer) elevated antioxidant activity compared to the all-*trans* isomer in vitro, demonstrated by α, α-diphenyl-β-picrylhydrazyl (DPPH) scavenging activity test.

### 4.1. Immune System and Inflammation

Astaxanthin plays a crucial role in boosting the immune response [133], and is a multi-target pharmacological carotenoid that helps in treating neurological disorders such as Parkinson’s disease (PD), Alzheimer’s disease, depression, aging, and brain and spinal cord injuries [134]. It is also reported to have anti-inflammatory effects in several conditions such as neurodegenerative disorders, gastrointestinal disease, diabetes, and renal inflammation, which makes it a potential anti-inflammatory agent [135]. It also helps in T helpers 1 cytokines production, such as IL-2 and Mouse interferon Y (IFN-Y) (Fakhri et al., 2018). It also reduces nuclear factor-κB (NF-κB) and other down-stream mediators such as IL-1β, interleukin (IL)-6, matrix metalloproteinase (MMP-9). In addition, it modulates phosphoinositide 3-kinases (PI3K)/Akt, ERK/MAPK, and the up-stream macrophage migration inhibitory factor (MIF) [136].

The cytokine storm is a serious immune dysregulation caused by cytokine overproduction, which often occurs during virus infection, organ transplant, autoimmune diseases, and immunotherapy, and it may ultimately result in death if untreated (Qin et al., 2020). Along with destroying the invaded pathogen (mostly virus), the dysfunctional immune response results in diffusive alveolar damage and pulmonary oedema (Tay et al., 2020). High levels of inflammatory factors such as tumor necrosis factor-alpha (TNF—α) and interleukin-6 (IL-6), cause respiratory distress. These inflammatory factors can be controlled with the help of astaxanthin. Astaxanthin regulates the cyclooxygenase-2 (COX-2) pathway and suppress cytokines and other inflammatory agents (IL-6 and TNF—α).

### 4.2. Treatment of Skin Conditions and Protection of Eyes Health

Exposure of skin to ultra-violet radiation increases the risk of ROS level in the skin resulting in increased oxidative stress that triggers multiple oxidative actions such as damage to DNA. This can damage the skin and its physiological functions [137]. UV radiation induces skin cancer by initiating a chain reaction resulting in the generation of peroxides and other free radicles from lipids, and damaging DNA, causing the development of cancer cells [121]. Studies have shown that oral administration and absorption of astaxanthin, and its subsequent accumulation in tissues, protect the skin from radiations, aging and skin diseases. Different chemical forms of astaxanthin are responsible for its function. 13-*cis* astaxanthin had greater accumulation in the skin and eyes of rodents than the all-*trans* and 9-*cis* isomers after two weeks of continuous administration (10 mg/mL) [138]. Moreover, studies have reported that if astaxanthin is included in a regular diet it has the potential to enhance skin quality, increase skin elasticity, and reduce facial wrinkles and pigmentation [139].

Even though the function of the eyes is the absorption of light, long-term exposure of light may cause oxidative stress causing destructive structural and functional changes in the lens, retina and eye tissues [140]. Moreover, long-term exposure of light to eyes may induce and increase ROS level, which can activate cellular pathways associated with inflammation. It has been demonstrated that astaxanthin can cross the blood-brain barrier and accumulate in the retina of animals [141]. Therefore, the consumption of astaxanthin can help maintain eye health by reducing oxidative inflammation and asthenopia by improving retinal blood flow. A study conducted by Harada et al., [142] found that corneal tissues of mice that were affected with photokeratitis were healed, and inflammation was decreased, when astaxanthin nano powder was given orally. This study suggested that the inflammation reduction was due to reduction in the expression of cyclocxygenase-2 (COX-2), phosphorylated inhibitor of κB-α (p-IκB-α), tumor necrosis factor-α (TNF-α), and CD45 in the corneal tissue. Astaxanthin can enhance eye blood circulation and help in curing the eyestrain [139,143].

## 5. Safety Measures to Fight against Infectious Diseases Using Astaxanthin

The broad range of therapeutic advantages of astaxanthin make it a potential agent in the regulation of the host immune system. Numerous studies have revealed the potential of astaxanthin in healthcare and in fighting certain diseases as an effective antioxidant, anti-inflammatory agent, immune system modulator and glucose-level lowering agent. Studies have reported that astaxanthin supplementation lowers oxidative biomarkers such as MDA and isoprostanes and increased antioxidant capability, which helped in decreasing low density lipoprotein (LDL) oxidation and alleviated lipid peroxidation [111,128,129]. These supplements are also known to reduce the plasma levels of 12 and 15 hydroxy fatty acids in males in about three months [144]. Astaxanthin supplementation modulates the JAK (Janus Kinase)/STAT3 (Signal Transducers and Activators of Transcription) signaling pathway. JAK kinases are a family of tyrosine kinases that mediate the signaling by tyrosine phosphorylation of various proteins and receptor chains bound and dimerized by STAT for their translocation to the nucleus for activation or inactivation of transcription. Astaxanthin also regulates the human immune system by stimulating mitogen-induced lymphocyte proliferation, delaying hypersensitivity, and increasing levels of natural killer cell cytotoxicity and the total count of T-cells and B-cells in the peripheral blood [133]. Detailed studies of astaxanthin on different animal models targeting specific genes for therapeutic study is listed in Table 2. Astaxanthin is also reported to be an IgM and IgG stimulant in mouse spleen cells and human immunoglobulins in human cells. Studies in various animal models have demonstrated the ability of astaxanthin to activate the forkhead box O3 gene (*FOXO3*) that controls cell fate, metabolism, resistance to DNA damage, autophagy and apoptosis [145,146]. It is also responsible for regulating aging and age-related diseases, such as cardiovascular diseases, diabetes, cancers and other neurodegenerative diseases [145].

## 6. Future Perspectives of Astaxanthin as a Novel Drug

Research and development processes for new drugs represent a continuous fight against infectious diseases. Epidemic situations create pressure in the search for new drugs that do not generate side effects [171]. Many proof-of-concept studies have been undertaken, but controlled trials are necessary to establish the effectiveness of astaxanthin in the treatment of numerous diseases [172]. Currently, researchers are conducting clinical trials, investigating the remarkable effects of astaxanthin on inflammation and oxidative stress.

In the future, astaxanthin may play a role as significant as statins in the treatment and possible prevention of cardiovascular, pulmonary and neurogenerative diseases [173], although its popularity is limited due to its instability at high temperatures, under low pH and illuminated conditions, its hydrophobic nature, and limited bioavailability [111]. Astaxanthin applications are limited in biomedical applications due to its unstable nature, but methods such as encapsulation can be useful to preserve the biochemical activity of this biomolecule for longer periods. A lot of research has been carried out on its biochemical, biophysical, and molecular properties, but its transportation, metabolism and interaction studies need to be explored in detail [174]. Astaxanthin has great health benefits against many diseases when obtained from natural sources. Innovation towards the development of biocompatible, low-cost extraction methods is required for sustainable use of astaxanthin in the nutraceutical sector.

## 7. Conclusions

Astaxanthin is a super ketocarotenoid with unique chemical isomerization in different source organisms. However, astaxanthin from *Haematococcus lacustris* is most widely cultivated because of its safe and strong antioxidant nature. *Haematococcus lacustris* can be mass cultivated and to produce a novel drug and dietary supplement if the cost of its production can be reduced using different techniques and strategies. Further the bioavailability of astaxanthin dosage is an important factor that requires a deeper study and extensive research if it is multifunctional applications in different pharmaceutical, nutraceutical, cosmetic and dietary industries are be considered. Development of innovative drugs such as natural astaxanthin from microorganisms, and especially from *Haematococcus lacustris*, can lead to solutions for enormous scientific, clinical, and societal problems. Outbreaks of infectious diseases have created major public health emergencies globally. Sustained and consistent research is required to enhance our knowledge of key aspects of viral pathogenesis that can lead to improved precautionary and therapeutic policies.

## Figures and Tables

**Table 1:** Chemicals/factors induced astaxanthin biosynthesis and accumulation by the upregulation of the carotenogenic genes in *Haematococcus lacustris*.

Factors/Chemicals	Astaxanthin Biosynthesis and Gene Expression	Reference
Salicylic acid and jasmonic acid	Up-regulation of carotenogenic genes of *psy*, *pds*, *zds* and *crtR-B*	[81]
Salicylic acid	Up-regulation by *ipi-1*, *ipi-2*, *psy*, *crtR-B*, *bkt* and *crtO* genes at transcriptional level. *lyc* gene at post-transcriptional level and *pds* gene at both.	[82]
Potassium iodide and high light	Upregulation of transcription levels of 15-cis-phytoene/all-transphytoene synthase gene (*CrtB*) and 15-cis-phytoene desaturase gene (*PDS*).	[78]
Trisodium citrate	Up-regulation and the expression of genes encoding beta-ring hydroxylase (*LUT5*), beta-carotene/zeaxanthin 4-ketolase (*CrtW*) and beta-carotene 3-hydroxylase (*CrtZ*).	[83]
CO_2_	Upregulation of genes involved in β-carotene biosynthesis such as *PSY*, *ZDS* and *lcyB* and β-carotene conversion like *crtZ*.	[84]
High light stress	Up-regulation and the expression of genes in MEP and astaxanthin biosynthesis pathway genes (*ISPF, GGPS*, *PDS, CrtW* and *CrtZ*) and concurrent down-regulation the expression of *SPS*, *CHLP* and *CrtL-e*.	[85]
Low temperature plasma	Expression of *IPT9* and *CYP735A1* involved in zeatin synthesis, amidase gene (*AMI1*), aldehyde dehydrogenase gene (*ALDH7A1*) in the indole-3-acetic acid (IAA) synthesis pathway, acyl-CoA oxidase gene (*ACX1*) in methyl Jasmonates synthesis pathway	[86]
High intensity blue and white LED light	Under white light upregulation of transcripts of astaxanthin biosynthesis genes *psy*, *crtO*, and *bkt2*. and under blue light upregulation of genes *psy*, *lcy*, *crtO*, and *crtR-B*.	[87]
Jasmonic acid	Astaxanthin biosynthesis up-regulation by *psy*, *pds*, *crtR-B*, *lyc*, *bkt2* and *crtO* at the transcriptional level and *ipi-1*, *ipi-2* at both transcriptional and post-transcriptional levels.	[88]
Blue, white, and red light	Blue light receptor gene upregulation of the biosynthesis pathway genes *psy* and *pds*, as well as *dgat1* and *dgat2d*.	[87]
Melatonin	Upregulation of the major metabolites of the TCA cycle and the GABA shunt.	[89]
Melatonin + 3-methyladenine	The gene encoding lycopene β-cyclase (*LCY*), which catalyses lycopene to β-carotene which acts as the direct precursor for the accumulation of astaxanthin, which is further catalysed by β-carotene hydroxylase (*CHY*).	[90]
Melatonin + Calcium	Up-regulation and the expression levels of astaxanthin biosynthetic genes (*lcy*, lycopene β-cyclase; *bkt*, β-carotene ketolase; *chy*, β-carotene hydroxylase)	[54]
Acetate and Fe^2+^	*PDS*, *crtISO*, *LUT1*, *LUT5*, *lcyB*, *lcyE*, *crtZ*, *CCD8*, *ZEP*	[91]
Under the monochromatic red (660 nm) or blue (450 nm) light-emitting diode (LED) irradiation	Upregulation and expression of gene *IPP* (encoding isopentenyl pyrophosphate), *PSY* (encoding phytoene synthase), *PDS* (encoding phytoene desaturase), *LYC* (encoding lycopene beta cyclase), *BKT* (encoding beta-carotene ketolase), *CHY* (encoding carotenoid hydroxylase), and *CBR* (encoding carotene biosynthesis-related protein (chlorophyll a-b binding protein)	[92]
Disodium 2-oxoglutarate (2-OG-2Na)	Up-regulation of genes *ipi, bkt* and *crtR-b*.	[93]
Sucrose	The expression of genes encoding phytoene synthase (*CrtB*), beta-carotene/zeaxanthin 4-ketolase (*CrtW*, BKT) and beta-carotene 3- hydroxylase (*CrtZ*).	[83]
Blue light and salicylic acid	Under blue light the expression of *fpps* was significantly regulated, and blue light reduced the expression of genes involved in astaxanthin synthesis.	[94]
Na_2_ WO_4_	Expression of *ipi*, *psy* and *bkt* for astaxanthin biosynthesis	[95]

Note: *PSY: Phytoene synthase; PDS: phytoene desaturase; ZDS: zeta-carotene desaturase; crtR-B: β-carotene hydroxylase; BKT: β-carotene ketolase; beta-ring hydroxylase (LUT5); ZDS: zeta-carotene desaturase; LcyB: lycopene beta-cyclase; MEP: methylerythritol 4-phosphate; PDS Phytoene desaturase; LYC: Lycopene beta cyclase; CHY: carotenoid hydroxylase; CBR: Carotene biosynthesis-related protein SPS: Sucrose phosphate synthase; CHLP: Chloroplastic; Lcy: Lycopene; TCA cycle: Tricarboxylic acid cycle; EMP pathway: Embden-Meyerhof-Parnas; PPP: Pentose phosphate pathway; IPP: Isopentenyl pyrophosphate; beta-carotene 3- hydroxylase (CrtZ); crtISO: Carotenoid isomerase; LUT1: Long undecoded transcript isoform-1; LUT5: Long undecoded transcript isoform-5; lcyB: Lycopene β cyclase; lcyE: Lycopene epsilon cyclase; crtZ: Beta-carotene hydroxylase; CCD8: Carotenoid cleavage dioxygenase 8; ZEP: Zeaxanthin epoxidase; ISPF: Isoprenoid F; GGPS: Geranylgeranyl pyrophosphate synthase.*

**Table 2:** Therapeutic actions of astaxanthin at different concentration on different gene markers in different test animals.

Test Parameter	Model	Astaxanthin Concentration	Target Gene/Biomarker	Reference
Anti-inflammation	Mice	25 mgkg^−1^ day^−1^	*NF-κB*, *TNF-α*	[147]
	Rats	1, 10 or 100 mgkg^−1^	*TNF-α*, *PGE2*, *IL-1βp-IKKα*, *p-IκBα*	[148]
	BV-2 cells	50 µM	*NF-κ* *B p65*, *IL-6*, *MAPK*	[149]
	Male Balb/c mice	50 mgkg^−1^	*Nrf2*, *NLRP3*, *IL-1β* *, * *IL-18*	[150]
	Male ICR mice	5 mgkg^−1^ day^−1^	*IL-1β*, *IL-6*, *TNF-α*	[151]
	BALB/c female mice	10 or 40 mg d^−1^	*IL-2 and IL-10*, *IFN-γ*	[152]
	HR-1 mice	10 µg or 20 µgcm^−2^	*NF-κ* *B*, *IL-1β* *, * *IL-6*, *TNF-α* *, * *IgE*, *COX-2*, *iNOS*	[153]
	Balb/cA mice	200 mg kg^−1^ body weight day^−1^	*IL-2*, *IFN-γ*, *IL-4*	[154]
	BALB/c mice	1 µL drop of 5 µM	*PI3K/Akt*, *HMGB1*, *TNF-α*, *IL-1β*	[155]
Antioxidant	Human	6 or 12 mgd^−1^	*PLOOH*	[156]
	PC12 cells	5*, *10*, *20 µM	*NOX2*, *Sp1/NR*, *NFR2*, *HO-1*	[157]
	Mice	2 mg kg^−1^	*APOP*, *SOD*, *GSH*, *MDA*	[158]
	PC12 cells	0.1 µM	*NF-κ* *B*, *Bax*, *IL-1β*, *TNFα*	[159]
	Primary hippocampal neurons	0.1 µM	*RyR2*, *NFATc4*	[160]
	SH-SY5Y cells	100 nM	*CYTc*, *PARP*	[161]
	Motor neurons	100 nM	*SOD1*	[162]
Ocular health	C57BL/6J mice	1/0.1/ 0.01 ng ml^−1^ by eye drop	*NF-κB*	[163]
	Db/db rats	25/5 mg kg^−1^ (oral gavage)	*8-OHdG*, *SOD*, *MDA*	[164]
	C57BL/6J mice	50 mg.kg^−1^	*Bax*, *Bcl-2*, *Nrf2. HO1*, *ROS*	[165]
	Wistar rats	0.6/3 mg kg^−1^	acrolein*, 8-OHdG*, *NO*, *MCP-1*, *ICAM-1*	[166]
	ddY mice	100 mg kg^−1^	*4-HNE*, *8-OHdG*	[167]
Neuroprotective	Rats	10 mg kg^−1^	*MVA*, *Nef2*, *SOD*	[168]
	SH-SY5Y cells and Rats	10 to 50 µM (cells) 30 mg kg^−1^ (rats)	*HSPs*, *iNOS*	[162]
	Mice	20 mg kg^−1^	*SOD*, *GHS*, *Casp3*, *Cyt C*	[169]
Alzheimer’s Disease	Wistar rats	10 mg kg^−1^ body weight	Oxidative markers	[170]

Note: *NF-Κb: Nuclear factor kappa B subunit 1; TNF-α*: Tumor necrosis factor alpha; *PGE: Prostaglandin E synthase; IL-1βp*-Interleukin (IL)-1β-; *IKKα*: inhibitor of nuclear factor kappa-B kinase subunit beta; *p-IκBα*: nuclear factor of kappa light polypeptide gene; *IL-6*: Interleukin-6; *MAPK*: Mitogen-activated protein kinase; *NLRP3*: Nucleotide binding domain, leucine rich containing family domain containing 3; *IL-18*: Interleukin-18; *IL-2*: Interleukin-2; *IL-10*: Interleukin-10; *IFN-γ*: Interferon gamma; *IgE*: Immunoglobulin E; *COX-2*: Cyclo-oxygenase 2; *iNOS*: Inducible nitric oxide synthase; *PI3K/Akt*: phosphoinositide-3-kinase–protein kinase B/Akt; *HMGB1*: High mobility group box protein 1; *PLOOH*: Phospholipid hydroperoxide; *NOX2*: phagocyte NADPH oxidase; *Sp1/N*: Transcription factor Sp1 N terminal; *NFR2*: Nuclear factor erythroid factor 2 related factor; *HO-1*: Heme oxygenase 1; *APOP*: Amyloid Beta Precursor Protein; *SOD*: Superoxide dismutase; *GSH*: Glutathion; *MDA*: Malondialdehyde; *RyR2*: Ryanodine receptor 2; *NFATc4*: Nuclear factor of activated T cells 4; *CYTc*: Cytochrome c; *PARP*: Poly(ADP- ribose) polymerase 1; *SOD1*: Superoxide dismutase 1; *8-OHdG*: 8-hydroxydeoxyguanosine; *NO*: Nitric oxide; *MCP-1*: Monocyte chemoattractant protein-1; *ICAM-1*: Intercellular adhesion molecule 1; *4-HNE*: 4-hydroxynonenal; *MVA*: Mevalonate pathway; *Nef2*: nuclear factor erythroid 2–related factor 2; *Casp3*: Caspase-3; *Cyt C*: Cytochrome C.

## Data Availability

Not applicable.
